# An Acute Bout of Endurance Exercise Does Not Prevent the Inhibitory Effect of Caffeine on Glucose Tolerance the following Morning

**DOI:** 10.3390/nu15081941

**Published:** 2023-04-18

**Authors:** Karoline T. Fenne, Matthieu Clauss, Daniela Schäfer Olstad, Egil I. Johansen, Jørgen Jensen

**Affiliations:** 1 Department of Physical Performance, Norwegian School of Sport Sciences, P.O. Box 4014, Ullevål Stadion, 0806 Oslo, Norway; 2 Polar Electro Oy, 90440 Kempele, Finland

**Keywords:** insulin sensitivity, diabetes, lactate, blood pressure, heart rate variability

## Abstract

Caffeine reduces glucose tolerance, whereas exercise training improves glucose homeostasis. The aim of the present study was to investigate the effect of caffeine on glucose tolerance the morning after an acute bout of aerobic exercise. Methods: The study had a 2 × 2 factorial design. Oral glucose tolerance tests (OGTT) were performed after overnight fasting with/without caffeine and with/without exercise the evening before. Eight healthy young active males were included (Age 25.5 ± 1.5 years; 83.9 ± 9.0 kg; VO_2max_: 54.3 ± 7.0 mL·kg^−1^·min^−1^). The exercise session consisted of 30 min cycling at 71% of VO_2max_ followed by four 5 min intervals at 84% with 3 min of cycling at 40% of VO_2max_ between intervals. The exercise was performed at 17:00 h. Energy expenditure at each session was ~976 kcal. Lactate increased to ~8 mM during the exercise sessions. Participants arrived at the laboratory the following morning at 7.00 AM after an overnight fast. Resting blood samples were taken before blood pressure and heart rate variability (HRV) were measured. Caffeine (3 mg/kg bodyweight) or placebo (similar taste/flavor) was ingested, and blood samples, blood pressure and HRV were measured after 30 min. Next, the OGTTs were initiated (75 g glucose dissolved in 3 dL water) and blood was sampled. Blood pressure and HRV were measured during the OGTT. Caffeine increased the area under curve (AUC) for glucose independently of whether exercise was done the evening before (*p* = 0.03; Two-way ANOVA; Interaction: *p* = 0.835). Caffeine did not significantly increase AUC for C-peptides compared to placebo (*p* = 0.096), and C-peptide response was not influenced by exercise. The acute bout of exercise did not significantly improve glucose tolerance the following morning. Diastolic blood pressure during the OGTT was slightly higher after intake of caffeine, independent of whether exercise was performed the evening before or not. Neither caffeine nor exercise the evening before significantly influenced HRV. In conclusion, caffeine reduced glucose tolerance independently of whether endurance exercise was performed the evening before. The low dose of caffeine did not influence heart rate variability but increased diastolic blood pressure slightly.

## 1. Introduction

Caffeine is a common constituent in many food items, and most adults in the USA and European countries consume caffeine on a daily basis [1,2]. Caffeine is an ergogenic substance [3,4], but caffeine has many other effects and reduces glucose tolerance [5,6,7]. Despite the widespread use of caffeine, studies exclude intake of caffeine before evaluating the effect of exercise on glucose metabolism [8,9,10,11]. Therefore, the effect of caffeine on glucose metabolism after exercise training remains unknown.

It is well-documented that caffeine reduces glucose tolerance, and intake of 2–3 cups of coffee increases plasma caffeine concentrations sufficiently to reduce glucose tolerance [5,12]. Moreover, the increase in glucose response during an OGTT after caffeine intake is paralleled with elevated insulin and C-peptide responses [5,6,13,14]. These data suggest that caffeine reduces insulin sensitivity, and indeed, it has been shown that caffeine reduces insulin sensitivity in humans [15,16]. Caffeine reduces glucose tolerance in both healthy populations and type 2 diabetics [7,15,17,18].

The pharmacological actions of caffeine (1,3,7-trimethylxantine) are well described [12]. Caffeine blocks adenosine receptors and PI 3-kinase and inhibits glycogen phosphorylase and phosphodiesterase (PDE) [12,19,20]. The inhibitory effect of caffeine on PI 3-kinase activity and insulin-stimulated glucose is an attractive mechanism to suggest explaining caffeine-mediated insulin resistance [19,21], but the physiological concentrations of caffeine in humans are too low to inhibit PI 3-kinase [12,22,23]. Moreover, caffeine reduces insulin-stimulated glucose uptake in humans without impairing insulin signaling [16], and the intracellular mechanisms by which caffeine reduces insulin sensitivity remain elusive.

Muscle contraction *per se* stimulates glucose uptake acutely [21,24,25,26,27] and increases insulin sensitivity afterwards [28,29,30], but the mechanisms are not well understood. Interestingly, caffeine reduces contraction-stimulated glucose uptake in skeletal muscles [21], despite muscle contractions stimulating glucose uptake independent of PI 3-kinase [27], suggesting that caffeine regulates glucose uptake via still unknown mechanisms. Exercise training improves insulin sensitivity and glucose tolerance [9,10,11]. Glucose tolerance can even be increased many hours after a single bout of exercise [31,32]. However, not all studies have shown increased glucose tolerance after training [33], and the reason for this discrepancy needs clarification. Interestingly, we found that an acute bout of exercise increased glucose tolerance in people on normal diet but not after 3 weeks on a high fat diet [31]. Moreover, it has been shown that the acute effect of exercise diminishes after training [34], and a period with very intense training can reduce glucose tolerance [35].

Caffeine is often used by athletes, and plasma glucose increases more quickly after exercise when caffeine is ingested together with carbohydrates [36,37]. However, the effect of caffeine on glucose tolerance many hours after training has not been studied. The mechanisms by which caffeine influences glucose tolerance are particularly interesting because epidemiological studies have shown that caffeine and coffee intake reduce the risk of developing type 2 diabetes [38]. Although it is documented that exercise improves metabolic regulation and fat loss [39], the interaction between caffeine and exercise on glucose tolerance remains unknown.

Caffeine intake in the morning is common, and it will therefore be important to clarify if exercise the days before prevents the reduction in glucose tolerance caused by caffeine. Therefore, the aim of the present study was to test the hypothesis that caffeine does not reduce glucose tolerance the day after a bout of endurance exercise. Caffeine increases blood pressure [40], but the effect of caffeine on heart rate variability during an oral glucose tolerance has not been clarified. Therefore, we investigated the effect of caffeine on blood pressure, heart rate (HR) and heart rate variability (HRV) during the OGTT the morning after a bout of exercise.

## 2. Materials and Methods

### 2.1. Subjects and Approvals

Eight healthy highly-trained males participated. Physical characteristics of the subjects were as follows (mean ± SD): age 25.0 ± 1.5 years; weight 83.2 ± 9.0 kg; height 179.8 ± 7.0 cm; body mass index 25.6 ± 1.8 kg/m^2^; maximal oxygen uptake (VO_2max_) during cycling 54.3 ± 7.0 mL‧kg^−1^‧min^−1^; number of training hours per week 7.3 ± 2.9 h. The study was approved by the Ethics Committee at Norwegian School of Sport Sciences (#94-090519) and registered at the Norwegian Center of Research Data (NSD). Subjects gave their written consent after being informed about the purpose of the study and the risk involved. 

### 2.2. Design/Experimental Procedures

**Figure 1:**
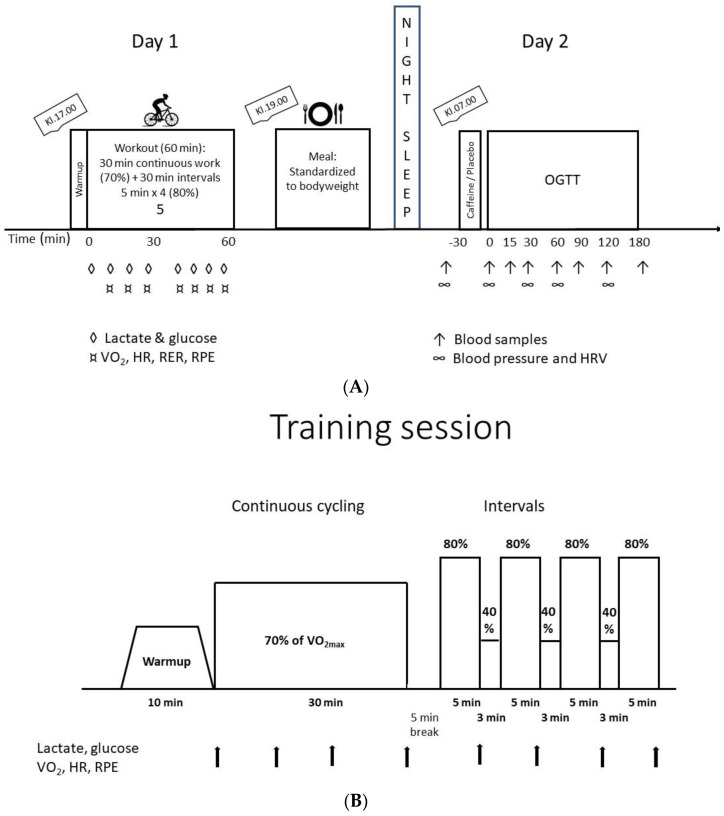
Schematic overview of the design study. (**A**) Schematic overview of the two intervention days completed four times. (**B**) Schematic overview of the acute bout of exercise completed the evening before the OGTT. Abbreviations to (**A**): OGTT: oral glucose tolerance test; VO_2_: oxygen uptake; HR: heart rate; RER; respiratory exchange ratio; RPE: rate of perceived exertion. Abbreviations to (**B**): VO_2max_: maximal oxygen uptake; VO_2_: oxygen uptake; HR: heart rate; RPE: rate of perceived exertion.

The study was randomized, double-blind and controlled, with 2 × 2 factorial crossover design. The subjects performed four oral glucose tolerance tests (OGTT) in total, with and without standardized exercise the day before and with and without caffeine intake 30 min prior to drinking the glucose solution (Figure 1A). The OGTTs were separated by one week.

### 2.3. Pre-Tests: Incremental Test and VO_2max_ Test

Before the intervention, participants completed an incremental test, and maximal oxygen uptake (VO_2max_) was measured. At the incremental test, participants cycled at a minimum of 4 loads for 5 min on a Lode ergometer bike (Lode Excalibur Sport, Lode B.V., Groningen, Netherlands), and oxygen uptake was measured with Oxycon Pro (Jaeger Instr., Hamburg, Germany). At the incremental test, the load increased by 25 W after each load until capillary lactate reached 4 mM, as described [41]. At each load, VO_2_ was measured from 2–4 min, and the rate of perceived exertion (RPE; Borg scale) and capillary lactate were measured (Biosen C-Line, EKF—diagnostic GmbH, Dümmer, Germany). Heart rate was recorded continuously with a H10 strap, and data were collected with Polar RS800CX (Polar Electro Oy, Kempele, Finland). The rate of perceived exertion was evaluated with the 6–20 Borg scale [42]. Participants maintained a cadence of 80–90 RPM.

After the incremental test, the participants cycled 10 min at self-selected intensity or rested before completing the VO_2max_ test. The VO_2max_ test started at a load 50 Watt below the highest load at the test to establish the relationship between load at VO_2_. The load increased 25 W per min until voluntary exhaustion, and the subjects were unable to maintain a cadence of over 60 RPM. Oxygen uptake was measured continuously. The recommended cadence was 80–90 RPM. VO_2_ was measured over 30 s periods throughout the test, and the average of the two highest consecutive measurements was used as VO_2max_. 

### 2.4. Measurement of VO_2_, CO_2_, Lactate and Glucoses during the Bike Tests

Oxygen uptake was measured by an Oxycon Pro ergospirometer (Jaeger Instr., Germany). The subject breathed through a two-way valve with a mouthpiece (Hans Rudolph Instr., Shawnee, OK, USA), and the expired air entered the mixing chamber. The volume transducer was calibrated manually using a 3 L pump according to the description. The O_2_ and CO_2_ analyzers were calibrated against room air (20.93% O_2_/0.03% CO_2_) and gas of known composition (approx. 16% O_2_ and 6% CO_2_.). The supplier stated a measurement uncertainty of 0.04% and 0.01% for the O_2_ and CO_2_ analyzers (Erich Jager GmbH, Hoechberg, Germany). 

Capillary blood for lactate measurements was taken from a fingertip after the tip was washed with sterile water and punctured with a disposable needle (Accu-Check, Safe-T-Pro Plus, Mannheim, Germany). The first drop of blood was wiped off, and a capillary tube was filled with blood and placed in an Eppendorf tube containing hemolyzing fluid in a ratio of 1:51 (Biosen C-Line, EKF—diagnostic GmbH, Germany). The tubes were turned end over end several times, and 20 μL was injected into the analyzer (Biosen C-Line, EKF—diagnostic GmbH, Germany). The instrument was calibrated with 12 mmol/L standard fluid before each test. The linearity was checked with 3 mmol/L and 15 mmol/L standard liquids. Coefficient of variation was <1.5% at 12 mmol/L. 

### 2.5. Registration of Meals, Physical Activity and Caffeine Intake

Participants registered food intake for the last 24 h before the first test day. The participants repeated the same diet before all tests. Participants were encouraged not to exercise the last 24 h before the tests. However, light exercise was allowed for some participants, and similar exercise sessions should have been repeated before all test.

The participants were asked to refrain from caffeine and nicotine for the last 24 h before the oral glucose tolerance test (OGTT). Based on the questionnaire, six out of eight subjects were considered caffeine users (consumed more than two cups of caffeinated coffee or tea daily or more than two caffeine-containing energy drinks per day). 

### 2.6. Standardized Exercise 

The participants completed a standardized bout of exercise at 17:00 h before two of the OGGTs. The load for each subject during the standardized workout was calculated based on the oxygen uptake on the incremental test and the VO_2max_ test, and the loads for the different intensities (40%, 70% and 80% of VO_2max_) were calculated using linear regression. The workout consisted of 30 min continuous cycling at 70% of VO_2max_, followed by 30 min high intensity interval cycling (Figure 1B). The interval consisted of 4 × 5 min cycling at 80% of VO_2max_ with 3 min cycling at 40% of VO_2max_ between intervals. The subjects were asked to have a cadence of 80–90 RPM. 

VO_2_, HR, RPE and RER were measured at 10, 20 and 30 min during the continuous work. At the 5-min intervals, we measured VO_2_, HR, RPE and RER in the last 1 ½ minutes in each interval. 

**Figure 2:**
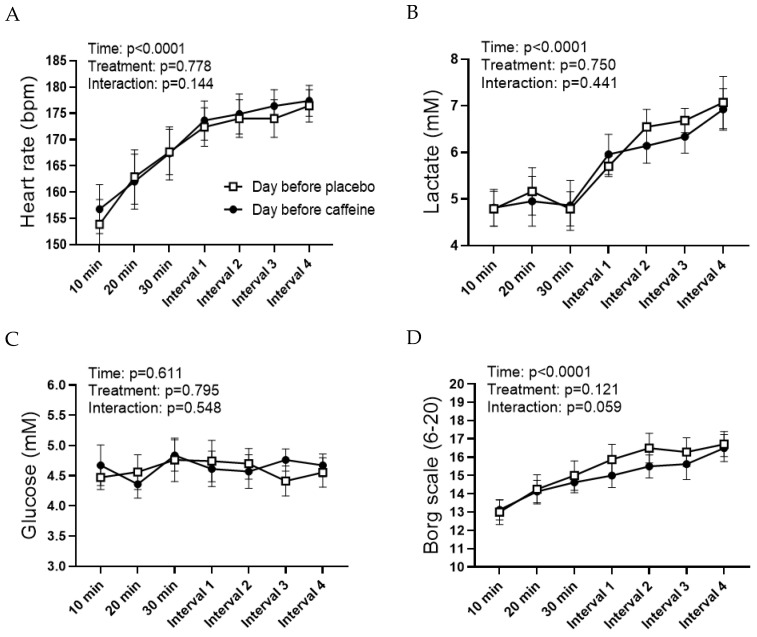
Metabolic responses to the exercise sessions. (**A**) Heart rate. (**B**) Capillary lactate. (**C**) Capillary glucose. (**D**) Perceived exertion according to the Borg scale. Data are mean ± SEM. *N* = 8 for all figures.

Capillary lactate and glucose were measured at time 0, 10, 20 and 30 min during the continuous work and after every 5 min at the interval work (Figure 2). The measurement of lactate is described above. Capillary glucose was measured at the same time (HemoCue Glucose 201 RT, Ängelholm, Sweden).

Energy utilization during the cycling sessions was calculated from the oxygen uptake, assuming 5 kcal per liter of oxygen [43]. Total oxygen uptake was calculated as averages at exercise intensities (70%, 80% and 40% of VO_2max_) and multiplied with the time carried out at the different intensities. Oxygen uptake was not measured at 40% of VO_2max_, and estimated values from the incremental test were used.

### 2.7. Standardized Meal

The participants received a standardized meal (10 kcal‧kg^−1^) 1 h after the exercise. The dinner consisted of pasta and meatballs in tomato sauce. Participants also ate a banana (~125 kcal) and a small protein pudding (Propud njie; 90 kcal) for dessert. The standardized dinner contained 51% carbohydrate, 28% fat and 18% protein and followed the dietary guidelines, which recommend 45–60% carbohydrate, 25–40% fat and 10–20% protein (Norwegian Directorate of Health). Energy was calculated from product declarations. The total energy content of the standardized dinner was 1047 ± 11 kcal. Participants drank water ad libitum.

### 2.8. Oral Glucose Tolerance Test (OGTT)

The participants arrived at the laboratory at 07.00 after an overnight fast. A venflon (BD Venflon TM Pro, Helsingborg, Sweden) was inserted in the antecubital vein, and a fasting blood sample was taken. Then the subjects ingested 3 mg‧kg^−1^ caffeine or a placebo drink. After 30 min, a new blood sample was taken (time 0) before the subjects ingested 75 g glucose dissolved in 3 dL of water. Blood samples were then taken at 15, 30, 60, 90, 120 and 180 min after glucose intake. After each blood test, the venflon was rinsed with saline solution (NaCl 0.9%, B. Braun Melsungen AG, Melsungen, Germany). Blood samples were obtained in EDTA tubes and kept on ice until centrifugation (3500× *g* at 4 °C for 10 min). After centrifugation, plasma was pipetted into Eppendorf tubes and stored at −80 °C. Serum blood samples for analysis of C-peptide were obtained at 0, 30 and 120 min. Serum tubes were allowed to coagulate for 30–40 min at room temperature and centrifuged (3500× *g* at 4 °C for 10 min). C-peptide was analyzed by Fürst Medisinsk Laboratorium (Fürst Medisinsk Laboratorium, Oslo, Norway). Venous blood glucose was analyzed immediately (HemoCue Glucose 201 RT; Ängelholm, Sweden). Due to problems with the venflon, glucose was measured in capillary blood for one of the participants. Area under the curve (AUC) for glucose and C-peptide was calculated with the trapezoid method. 

### 2.9. Blood Pressure, Heart Rate and Heart Rate Variability

Blood pressure, HR and HRV were measured before and during the OGTT. Blood pressure was measured 2–3 times per subject for each measurement (OMRON, HBP-1300, Professional BP Monitor, Hoffman Estates, IL, USA), and means were calculated. Measurements were done before intake of caffeine, before intake of glucose (t = 0) and 30, 60 and 120 min after glucose intake. HR and HRV were measured immediately after measurements of blood pressure. Data on heart rate were sampled at 1000 HZ with Polar V800 (Polar Electro Oy, Kempele, Finland) with a 6-min test: first 3 min sitting, then 3 min standing. For analysis of HRV, RR intervals were extracted and transferred to the software program Kubios-HRV, V2.1 (Department of Physics, University of Kuopio, Kuopio, Finland). Trend components were removed using the smoothness prior’s method (Lambda 500, frequency = 0.035 Hz), and signals were corrected with the Kubios artifact correlation filter. The applied filter was the lowest that removes all artifacts. The power spectral density of signals was estimated using Welch’s periodogram method. Seated and standing segments were separated and included for analysis if they contained at least 90% valid data—free from artifacts or ectopic heartbeats. Therefore, HRV could not be calculated in one of the subjects due to abnormal RRI (less than 90% valid data in Kubios HRV). From time-domain indexes, the following parameters were selected: mean heart rate (HR in bpm), mean value of RR intervals (RRI in ms), square root of the variance of the RRI (SDNN in ms), and the square root of the mean squared difference of successive RRI (RMSSD in ms). From frequency domain indexes: the ratio between low frequency power and high frequency power (LF/HF ratio) was selected and calculated. The frequency band for LF was 0.04 to 0.15 Hz, and for HF, 0.15 to 0.4 Hz. 

### 2.10. Statistical Analyses 

The results are presented as mean ± SEM. Area under the curve (AUC) for glucose and C-peptide were calculated according to the trapezoid method. Two-way ANOVA for repeated measurement was used to test effect of caffeine and exercise on glucose tolerance (AUC). Repeated measurements ANOVA was used to test the parameters during the standardized exercise and OGTT, with Sidak as the post hoc test. Three-way ANOVA for repeated measurements was used to test significance caffeine, exercise and OGTT on HRV and blood pressure. GraphPad Prism 8 program (GraphPad Software, La Jolla, CA, USA) was used for data analysis and graph design. The significance level was set at 5% (*p* < 0.05).

## 3. Results

The two training sessions completed the evening before the OGTTs were identical regarding load and duration. The session consisted of 30 min continuous cycling at 71.4 ± % of VO_2max_ (204 ± 4 W) followed by four 5 min intervals at 83.8% of VO_2max_ (242 ± 4 W) with 3 min at 40% of VO_2max_ between intervals. The heart rate increased from ~155 beats·min^−1^ after 10 min to ~172 beats·min^−1^ after 30 min cycling (Figure 2A). The mean heart rate at the intervals was ~175 beats·min^−1^. There were no differences in heart rate between the two sessions. (Figure 2A). Plasma lactate was ~5 mM during the first 30 min of cycling and gradually increased to ~7 mM during the interval part at both sessions (Figure 2B). Blood glucose remained at ~4.5 mM at both sessions (Figure 2C). RPE increased gradually during both sessions (Figure 2D). Calculated energy utilization at the sessions before the placebo and caffeine were 936 ± 16 kcal and 952 ± 18 kcal. There were no significant differences in any parameters at the sessions before OGTT with placebo and caffeine. 

The participants arrived at the laboratory at 7:00 am after an overnight fast, and glucose concentration was ~5 mM on all days (Table 1). Caffeine did not significantly influence plasma glucose, but glucose was higher 30 min after intake of both placebo and caffeine compared to arrival (Table 1). Fasting C-peptide at arrival did not differ between the four days (Table 1). 

**Figure 3:**
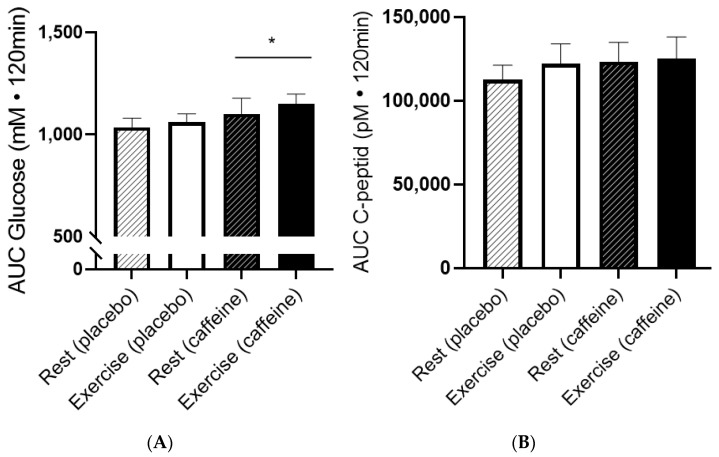
Effect of caffeine on glucose and C-peptide responses to OGTT on days with and without exercise the day before. (**A**) Area under curve (AUC) for glucose during the OGTT. (**B**) Area under curve for C-peptide during the OGTT. Data are mean ± SEM. *N* = 8. *: *p* < 0.05; Two-way ANOVA comparing AUC for glucose with and without caffeine.

Glucose increased rapidly after ingestion of 75 g glucose and peaked after 30 min. Caffeine increased AUC for glucose by ~7% (Figure 3; *p* = 0.027; Two-way ANOVA). There was no significant effect of exercise on AUC for glucose the evening before (Two-way ANOVA; *p* = 0.39) or interaction between intake and condition (*p* = 0.835). Exercise the evening before did not influence AUC for glucose, whether caffeine was ingested or not (Figure 3A). AUC for glucose inversely correlated with VO_2max_ in placebo the day after exercise (R^2^ = 0.603; *p* = 0.0235). 

During the OGTT, C-peptide increased to ~1200 pM after 30 min for all tests. There was a tendency for higher AUC for C-peptide after caffeine intake compared to placebo (Figure 3B; *p* = 0.096; Two-way ANOVA).

**Figure 4:**
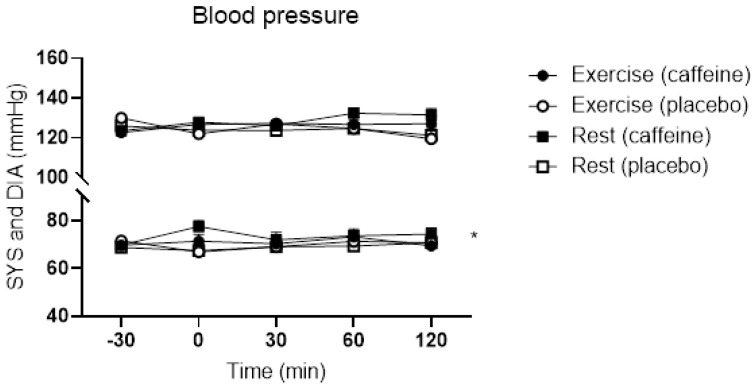
Blood pressure during the OGTT with and without caffeine intake before. OGTTs were conducted with and without an acute bout of exercise the evening before. Abbreviations: SYS: systolic blood pressure; DIA: Diastolic blood pressure. Data are mean ± SEM. *: *p* < 0.05; Two-way ANOVA comparing diastolic blood pressure with and without caffeine.

Systolic and diastolic blood pressure were normal and did not change during the OGTT. However, diastolic blood pressure was higher after intake of caffeine compared to placebo (Figure 4; *p* = 0.045; Two-way ANOVA). Systolic blood pressure tended to be higher (Figure 4; *p* = 0.075; Two-way ANOVA). There was no effect of caffeine on HR during the OGTT (Data not shown).

Heart rate variability did not change significantly during the OGTT, nor did caffeine have a significant effect on HRV (Table 2). 

## 4. Discussion

The main finding in the present study was that caffeine reduced glucose tolerance to a similar degree with and without exercise the evening prior to the glucose tolerance test. Moreover, intake of 3 mg/kg of caffeine did not significantly influence HRV but slightly increased diastolic blood pressure. Our data did not support our hypothesis that exercise could prevent (modifying effect) the reduction in glucose tolerance caused by caffeine.

In the present study, participants received 3 mg/kg of caffeine 30 min before the OGTT and AUC for glucose increased ~10% in young fit males. Most studies provided the participants with a higher amount of caffeine (typically ~5 mg/kg) and observed a larger increase in AUC for glucose [13,18,44,45]. Importantly, Beaudoin et al. established the dose–response relationship for caffeine-induced glucose tolerance by investigating the effect of 1, 3 and 5 mg/kg of caffeine in young healthy females and males [17]. Even the lowest dose of caffeine reduced glucose tolerance, and each mg/kg of caffeine increased AUC for glucose by 5–10% [17]. The fact that we observe a smaller effect increase in AUC for glucose compared to studies giving 5 mg/kg caffeine is therefore supported [17]. Moreover, our finding is supported by many other studies that have reported that caffeine or coffee reduces glucose tolerance in various cohorts, including type 2 diabetics [13,14,17,45]. 

Caffeine intake did not significantly increase AUC for C-peptide in the present study. Other studies have reported that AUC for C-peptide and insulin increases after intake of caffeine [6,14,44]. However, the increase in AUC for C-peptide increases dose-dependently after intake of caffeine, and Beaudoin et al. found that AUC for C-peptide increased 5.8% per mg/kg of caffeine [17]. In the present study, there was a tendency for increased AUC for C-peptide after intake of a low dose of caffeine (*p* = 0.096; 5.9% higher), which does not contrast other studies. The insulin response to an OGTT is also increased in both healthy participants and in type 2 diabetics [45]. The fact that caffeine increases AUC for both glucose and insulin indicates that insulin sensitivity is impaired.

The mechanisms by which caffeine reduces glucose tolerance remain unknown. It is well-documented that caffeine reduces insulin sensitivity [15,46,47], but insulin signaling was not impaired by the physiological concentration of caffeine [16]. High concentrations of caffeine effectively block insulin signaling and insulin-stimulated glucose uptake [21], but the plasma concentration after intake of 3 mg/kg body weight is only 20–30 µM [23]. Caffeine intake increases plasma adrenaline [15,48], but the increase in adrenaline is too low to explain the insulin resistance caffeine mediates [48]. Caffeine also increases plasma FFA [45], and the accumulation of intracellular acyl-CoA causes insulin resistance [49]. However, much higher concentrations of FFA, and for several hours, are required before insulin resistance develops in humans [50]. Furthermore, a higher amount of caffeine than provided in the present study seems required to increase fat oxidation during exercise [51]. Therefore, it seems unlikely that elevated FFA after caffeine intake mediated the reduction in glucose tolerance. Instead, it seems likely that the blockade of adenosine receptors contributes to the development of insulin resistance because adenosine receptors are the only signaling molecules with affinities for caffeine in low physiological concentrations [12]. There are four isoforms of adenosine receptors, and the A_2_-adenosine receptor seems to mediate the effect of caffeine on glucose tolerance [52,53].

Exercise improves insulin sensitivity and glucose tolerance [9,10,11,54]. Glucose tolerance can even be increased up to 14 h after a single bout of exercise [31,32]. In the present study, glucose tolerance was not elevated the day after a training session as expected. Interestingly, we found that an acute bout of exercise increased glucose tolerance in people on a normal diet but not after 3 weeks on a high fat diet [31]. Moreover, it has been shown that the acute effect of exercise diminishes after training [34]. Furthermore, a period with very intense training can reduce glucose tolerance [35], but although the exercise session in the present study resulted in high lactate, the participants were well-trained and tolerated the training well. 

The hypothesis that caffeine would not reduce glucose tolerance when a bout of exercise was performed the evening before the OGTT was not supported. Instead, caffeine increased AUC for glucose to a similar degree with and without exercise the day before (interaction *p* = 0.835). This is the first study to investigate the effect of caffeine on glucose tolerance the day after exercise. However, caffeine is often ingested by athletes, and the effect of caffeine on glycogen synthesis has been investigated. Importantly, caffeine does not seem to impair the rate of glycogen synthesis after exercise [36,55]. The effect of caffeine on glucose response after exercise was also investigated in these studies [36,55], but the data were inconclusive, and caffeine increased glucose response in one study [36] but not in another [55]. It is also noteworthy that intake of caffeine without glucose does not increase plasma glucose at rest despite higher levels being seen during exercise [3,56]. Importantly, for the first time, our data show that caffeine reduced glucose tolerance in healthy young males whether or not a single bout of endurance exercise was conducted 14 h before the glucose tolerance.

Epidemiological studies have shown that intake of coffee reduces the risk for development of type 2 diabetes [38,57,58,59], and the mechanism will be important to clarify. Coffee contains ~1000 different molecules, and many have antioxidant effects [60]. Interestingly, Battram et al. found that caffeine reduced glucose tolerance more than coffee containing the same amount of caffeine, and that decaffeinated coffee improved glucose tolerance compared to placebo [44]. There are also epidemiological data supporting other molecules than caffeine contribute to reducing the risk for type 2 diabetes [38,58]. Still, it is surprisingly that caffeine reduces the risk for the development of type 2 diabetes since acute intake of caffeine reduces glucose tolerance.

Caffeine slightly increased blood pressure, but HR and HRV were unchanged, and these effects were independent of a single bout of exercise the day before. The small increase in diastolic blood pressure after caffeine intake was expected [15,40]. The effect of caffeine on HR and HRV remains ambiguous and probably depends on intake dose. Most studies showed no effect on HR, while some studies reported increases or decreases following caffeine ingestion [40]. Similarly, most studies reported no effect of caffeine on HRV [61,62,63], while also an increase [64] or a decrease in HRV was reported [65]. Interestingly, ingesting 100 g of glucose increased HRV in pregnant women [66], and to our knowledge, this is the first study showing no effect of OGTT on HRV in young healthy males. Blood pressure, HR and HRV were not different the days after exercise compared to the days without exercise. HRV recovery from some bouts of exercise can take longer than 24 h [67,68,69]. Thus, the training stimulus of the exercise used in this study was not high enough to cause a change in cardiac autonomic modulation the following morning. This might be because the participants had a high fitness level and were, therefore, able to fully recover from the exercise with respect to cardiac autonomic modulation [69].

Strength and limitations: The study had a 2 × 2 factorial design, which is a strong design to test the effect of caffeine with and without exercise. It is also a strength that the four tests were conducted one week apart, allowing enough washout between tests. It is a strength that the participants completed the exercise session at the same time of the day and received a standardized meal after the exercise sessions and on control days the evening before all OGTTs. It is a weakness that only well-trained young men were included, which reduces generalization. Furthermore, it is a limitation that the number of participants was limited, which reduces statistical power. However, the inhibitory effect of caffeine with and without exercise the evening showed no interaction (*p* = 0.835). Therefore, an acute bout of exercise in the evening does not seem to influence the inhibitory effect of caffeine on glucose tolerance the following morning. 

In conclusion, the present study showed that caffeine reduced glucose tolerance in fasted conditions independently of whether exercise was performed the evening before or not. Therefore, the reduced glucose tolerance caused by caffeine, independent of exercise or not the preceding day, seems not to increase the risk of developing type 2 diabetes. The low dose of caffeine (3 mg/kg) did not influence heart rate variability but slightly increased diastolic blood pressure.

## Figures and Tables

**Table 1:** Blood glucose and serum C-peptide before the four oral glucose tolerance tests.

	Rest/PLA	Rest/CAF	Ex/PLA	Ex/CAF
Fasted glucose (mM)	5.0 ± 0.1	5.3 ± 0.1	5.2 ± 0.1	5.1 ± 0.1
Glucose PLA/CAF (mM) *	5.4 ± 0.1	5.4 ± 0.1	5.3 ± 0.1	5.6 ± 0.1
C-peptide (pM)	334 ± 18	300 ± 10	351 ± 28	301 ± 11

Data and mean ± SEM. Abbreviations: Rest/PLA and Rest/CAF: No exercise the days before and placebo or caffeine before OGTT, respectively. Ex/PLA and Ex/CAF: Exercise the days before and placebo or caffeine before OGTT, respectively. Glucose CAF/PLA: Blood glucose 30 min after intake of placebo or caffeine. *: Two-way ANOVA; Time effect (*p* = 0.019).

**Table 2:** Heart rate variability before intake of caffeine/placebo (t = −30 min), 30 min after caffeine intake (t = 0) and 30 min after intake of 75 g glucose for the OGTT.

	Rest + Placebo	Rest + Caffeine	Training + Placebo	Training + Caffeine
**Time −30 min**				
**Sitting**				
HF [bpm]	61 ± 2	63 ± 2	65 ± 1	65 ± 1
RRI [ms]	1019.8 ± 29.2	979.5 ± 25.2	946.8 ± 16.3	951.3 ± 19.2
SDNN [ms]	82.97 ± 5.63	70.21 ± 4.56	76.02 ± 3.93	74.07 ± 5.54
LF/HF	4.84 ± 0.40	6.16 ± 1.03	6.56 ± 0.36	4.53 ± 0.39
**Standing**				
HF [bpm]	73 ± 2	77 ± 1	76 ± 1	77 ± 1
RRI [ms]	745.7 ± 48.9	790.6 ± 12.9	792.4 ± 9.3	785.3 ± 12.0
SDNN [ms]	68.08 ± 4.88	50.90 ± 1.58	56.36 ± 2.84	51.03 ± 3.74
LF/HF ratio	8.26 ± 1.10	12.51 ± 1.57	11.60 ± 1.41	9.22 ± 0.83
**Time 0**				
**Sitting**				
HF	57 ± 1	59 ± 2	61 ± 1	62 ± 2
RRI [ms]	1063.9 ± 20.1	1040.3 ± 29.,9	999.5 ±16.8	991.55 ± 31.5
SDNN [ms]	81.69 ± 3.86	84.59 ± 4.18	83.99 ± 5.63	84.25 ± 7.02
LF/HF ratio	3.93 ± 0.39	3.50 ± 0.27	3.95 ± 0.23	4.29 ± 0.62
**Standing**				
HF	69 ± 1	68 ± 1	71 ± 1	70 ± 2
RRI [ms]	883.5 ± 15.2	893.9 ± 19.1	833.6 ±14.9	884.9 ± 23.5
SDNN [ms]	81.30 ± 4.35	75.91 ± 2.54	65.70 ± 3.13	78.26 ± 5.17
LF/HF ratio	4.46 ± 0.31	4.44 ± 0.35	4.69 ± 0.27	4.85 ± 0.51
**Time 30 min**				
**Sitting**				
HF	59 ± 2	61 ± 2	63 ± 1	61 ± 1
RRI [ms]	1053.1 ± 30.7	1001.7 ± 26.1	964.9 ±14.3	998.61 ± 18.1
SDNN [ms]	80.40 ± 3.78	89.61 ± 7.07	88.79 ± 5.45	84.13 ± 4.23
LF/HF ratio	5.07 ± 0.68	4.52 ± 0.52	3.88 ± 0.20	3.72 ± 0.43
**Standing**				
HF	70 ± 2	71 ± 2	73 ± 1	70 ± 1
RRI [ms]	881.2 ± 22.1	848.6 ± 17.1	831.2 ± 12.1	867.6 ± 13.2
SDNN [ms]	64.59 ± 4.37	84.33 ± 6.56	69.23 ± 4.17	69.02 ± 2.45
LF/HF ratio	5.43 ± 0.53	5.63 ± 0.34	5.41 ± 0.48	7.34 ± 0.94

Data are mean ± SEM. Abbreviations: HF: heart rate; RRI: beat-to-beat interval between R-tops in QRS-complex; SDNN: Standard deviation for all RR intervals; LF/HF ratio: low frequency power [0.04–0.15 Hz]/high frequency power [0.15–0.04 Hz]). *n* = 7.

## Data Availability

Not applicable.
